# Communities of practice in the academic library: strategies for implementation

**DOI:** 10.29173/jchla29640

**Published:** 2022-12-01

**Authors:** Alex Goudreau

**Affiliations:** Science & Health Sciences Librarian University of New Brunswick Saint John, NB, Canada

Reale M. **Communities of practice in the academic library: strategies for implementation**. Chicago: ALA Editions; 2022. Softcover: 100 p. ISBN: 978-0-8389-3648-1. Price: USD$54.99. Available from: https://www.alastore.ala.org/copacad

If you would like to connect with colleagues in an intentional and meaningful way wherein you share knowledge, learn together, and work through problems, *Communities of practice in the academic library: strategies for implementation* is a good fit for you. Michelle Reale’s latest book takes readers through her experience starting a community of practice (CoP) during the Covid-19 pandemic. It makes a case for developing CoP as spaces for transformational change and personal growth while encouraging colleagues to look outward, be collaborative, and develop learning relationships with each other. Though her work is geared toward academic libraries, much of what she shares through stories and practical guidance will be applicable to a wide range of health library professionals.

Reale is a professor and an access and outreach librarian at Arcadia University in Pennsylvania who has written about teaching, mentoring, and being an embedded librarian. She situates her recent CoP experience within the wider literature about CoP and draws heavily on Wenger et al.’s work in *Cultivating communities of practice: a guide to managing knowledge*. As far as I know, Reale’s book is the only library-specific one on the topic, though a recent literature review by Louque (2021) provides other examples of academic libraries using CoP frameworks.

This is a short book at 100 pages, with nine chapters touching on what goes into creating a successful CoP (e.g. how to bring people together, how to think and learn together, and how to have conversations to really listen to each other). Some chapters are more a descriptive “how to” for starting your own CoP, with suggestions on the first meeting and what key components are essential (understanding the purpose of the group, drafting a statement of goals, etc.). Other chapters are good refreshers on cultivating a reflective practice and mindfulness. Reale describes how these individual practices can then benefit your CoP group. Overall, the chapters all feel “snack sized” with subheadings to make it easy to revisit certain sections as needed. Each chapter ends with a brief summary, points to ponder (reflective questions and activities), and references.

This is a well-researched book, and Reale’s interest in the topic comes across in her writing and references. She shares specific examples from her own CoP and candidly discusses its successes and failures to date. The book has an honest and open feel. It is written in a conversational, accessible style. Reale links CoP literature to the needs and settings of library professionals effectively. She encourages a different mindset, one where librarians move away from being insular and competitive, and away from constantly reinventing the wheel within the knowledge-hoarding world of academia (something that likely resonates with folks in hospital settings too). Instead, she advocates for creating a CoP to build a knowledge base that facilitates sharing and learning among colleagues. She describes how such a community should be informal with disagreements encouraged to get people out of their comfort zones. Though her frankness may turn some people off, I found it refreshing and a good reminder that we can change our own practice to figure out how to work better together.

There are times in the book where it felt like Reale had a window into my own thinking and past experiences. When she describes how the problem her CoP was working on started out as a shared frustration of not having true collaboration with faculty when teaching information literacy and wanting something beyond the literature or an individual colleague for help, I felt very seen. In the chapter “Keep talking, I’m listening”, Reale goes into detail about how to create this problem-solving space, describing something that I know would feel foreign to many peoples’ current work situations – a comfortable place for people to be themselves and share their thoughts openly without judgement in a way that is different from a regular meeting or working group. Many people probably assume that a CoP functions in a similar way to other types of meetings or collaborative work, but Reale demonstrates how it can be different and how to actually make space for true conversation. I found this chapter fascinating because it addresses some of the most challenging aspects of fostering a CoP: getting people to actually listen to each other, allowing for meandering conversations, and allowing people to express how they really feel. These aspects of fostering a CoP counter the ways we are used to functioning within a group.

What resonated most with me was how Reale came to realize something needed to change in her practice. Her description of meetings that focus on business items without making time for problem-solving conversations was very familiar. She notes how the absence of these conversations, combined with peoples’ inability to admit knowledge gaps or be vulnerable with colleagues, makes it difficult to come together for open knowledge sharing and learning. Open knowledge sharing and learning is something I very much want for myself and my colleagues. I have made failed attempts to create this kind of space in the past. I might have salvaged those failed attempts if this book had existed then, and I will now use it to help lay a better foundation for a future attempt.

Reale concludes the book by explaining that her goal was to write not just a detailed how-to-guide, but something more hopeful that people can adapt to their own settings when building a community focused on problem-solving. I think she succeeds in that respect, particularly by inspiring hope for my own practice to try again to develop a CoP at my workplace. Given how new Reale’s own CoP is, it is understandable some experiences likely have not occurred yet and thus she was not able to address certain topics I was interested in. For example, I would have liked guidance on how to maintain long-term interest and engagement of people in a CoP, particularly during busier times like a fall academic semester, or what to do when library administration starts to question the CoP’s value or tries to shut it down.

Overall, this book has provided me with specific ideas about how I could create a CoP. It has given me language to use when talking to others about the purpose of a CoP, and strategies to help get people in the right mindset to participate. I plan on revisiting different chapters when I am ready to get started and will suggest the book to interested colleagues. I would recommend it to any library professional who has an interest in being more collaborative, sharing knowledge, and learning from others to solve problems together.
